# Workflow and Short-Term Functional Outcomes in Simultaneous Acute Code Stroke Activation and Stroke Reperfusion Therapy

**DOI:** 10.3390/neurosci5030023

**Published:** 2024-08-22

**Authors:** Robert Joseph Sarmiento, Amanda Wagner, Asif Sheriff, Colleen Taralson, Nadine Moniz, Jason Opsahl, Thomas Jeerakathil, Brian Buck, William Sevcik, Ashfaq Shuaib, Mahesh Kate

**Affiliations:** 1 Department of Medicine, University of Alberta, Edmonton, AB T6G2B7, Canada; rsarmien@ualberta.ca (R.J.S.);; 2 College of Medicine, University of Saskatchewan, Regina, SK S4S 0A2, Canada; 3 Stroke Program, Edmonton Zone, Alberta Health Services, Edmonton, AB T6G2J3, Canada; 4 Department of Emergency Medicine, University of Alberta, Edmonton, AB T6G2T4, Canada

**Keywords:** simultaneity, triage, acute code stroke activations, acute stroke, acute reperfusion treatment, patient outcomes

## Abstract

The burden of simultaneous acute code stroke activation (ACSA) is not known. We aim to assess the effect of simultaneous ACSA on workflow metrics and home time at 90 days in patients undergoing reperfusion therapies in the emergency department. Simultaneous ACSA was defined as code activation within 60 min of the arrival of any patient receiving intravenous thrombolysis, within 150 min of the arrival of any patient receiving endovascular thrombectomy, within 45 min of the arrival of any patient receiving no reperfusion therapies (based on mean local door-to-needle and door-to-puncture times). Simultaneous ACSA was further graded as 1, 2 and 3. We assessed workflow metrics as door-to-CT (DTC) time, in minutes, and functional outcome as home time at 90 days. A total of 2605 patients were assessed as ACSA at a mean ± SD activations of 130.8 ± 17.1/month and 859 (33%) were simultaneous. Among all ACSA, 545 (20.9%) underwent acute reperfusion therapy with a mean age of 70.6 ± 14.2 years, 45.9% (n = 254) were female with a median (IQR) NIHSS of 13 (8–18). A total of 220 (40.4%) patients underwent simultaneous treatments. The median DTC time, in minutes, was prolonged in grade 3 simultaneous ACSA (18 (13, 28)) compared to non-simultaneous ACSA (15 (11, 21) β = 0.23, *p* < 0.0001). There was no difference in the median home time at 90 days between the simultaneous (58, 0–84.5 days) and non-simultaneous (54, 0–85 days) patients. Simultaneous ACSA is frequent in patients receiving acute reperfusion therapies. An optimal workflow in high-volume centers may help mitigate the clinical and system burden associated with simultaneity.

## 1. Introduction

Early reperfusion of the ischemic brain is the most effective means of improving functional outcomes in both anterior and posterior circulation ischemic strokes. Over the past decade, innovations in acute stroke therapies have resulted in the expansion of the therapeutic time window to include patients presenting up to 24 h from stroke onset, creating challenges from a system implementation perspective [1,2,3]. System reorganization at varying levels (pre-hospital care and notification, imaging, emergency department (ED) and stroke unit) has been required to optimize workflow for a round-the-clock assessment of stroke patients for intravenous thrombolysis (IVT) and endovascular thrombectomy (EVT) [4,5].

Delays in IVT or EVT are associated with reduced benefits from reperfusion treatment, worse functional outcomes and longer hospital stays. To rapidly identify patients for reperfusion treatment in the ED in a timely manner, many centers have developed an acute code stroke activation (ACSA) process. The ACSA is usually triggered by nursing staff at ED triage and alerts the stroke team (computed tomography technologists, stroke nurse and stroke fellow/resident/physician) of an incoming patient with a suspected stroke [6,7]. It is not uncommon for multiple patients with suspected stroke to simultaneously present to the ED either directly or via transfer from other hospitals. These simultaneous ACSA incidences may strain the acute stroke management workflow and the stroke team which is designed to deliver care for a single stroke patient at a time [8]. Simultaneity and overcrowding in the ED can increase door-to-imaging time [8], affect decision making, and increase the risk of medical errors [9]. Furthermore, concurrent demand for EVT may increase door-to-groin puncture (DTP) times [10]. The problem of simultaneous ACSA is further compounded by the ongoing overcrowding of the ED by non-stroke and stroke mimic presentations [11,12]. Adjusted treatment time (ED bed assignment to ED providers’ disposition decision time) for all neurological presentations of acuity level 2 is 66.7 min, 211.7 min and 497 min for the 10th percentile, 50th percentile and 90th percentile, respectively [13]. The burden of simultaneous ACSA and simultaneous stroke reperfusion therapies is not known. To understand the effect of simultaneity on the hospital system, assessing workflow metrics by using DTP, for example, will be helpful. Thus, we aim to assess the effect of simultaneous ACSA on workflow metrics and home time at 90 days in patients undergoing reperfusion therapies in the emergency department.

## 2. Materials and Methods

The local ethics committee approved the study protocol (protocol number Pro00124351). This is a retrospective chart review; hence, consent was not obtained. The study data will be available from the corresponding author upon reasonable request and with the permission of all contributing authors.

### 2.1. Study Design and Setting

**Figure 1:**
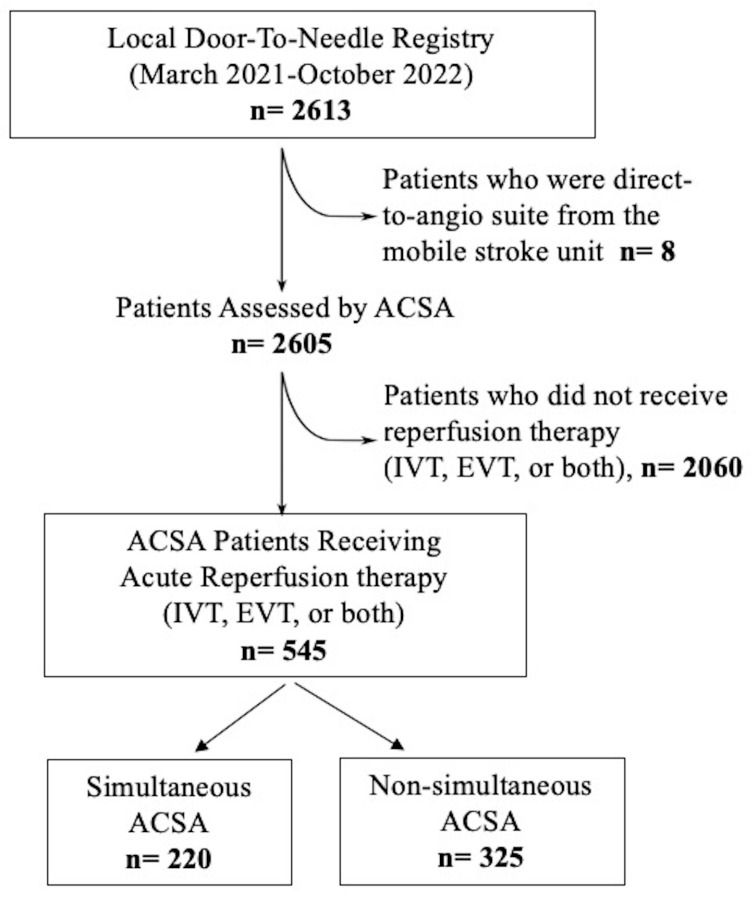
Study flowchart. ACSA: acute code stroke activation, IVT: intravenous thrombolysis, EVT: endovascular thrombectomy.

This is a retrospective analysis of a prospective local door-to-needle (DTN) quality improvement registry of acute stroke treatments and presentations to a comprehensive stroke center. To assess the overall burden of simultaneity, we included all patients who presented to the study hospital as an ACSA between March 2021 and October 2022. Patients directed to the angio suite from the mobile stroke unit were excluded. We further analyzed the effect of simultaneity on workflow metrics and home time by assessing those who received reperfusion therapies (Figure 1). The study site has three computed tomography (CT) scanners (one scanner in the ED and two elsewhere in the hospital), two EVT suites and one EVT nurse. Both stroke and non-stroke patients receive treatment in the study site’s ED. 

### 2.2. Code Stroke Workflow

**Figure 2:**
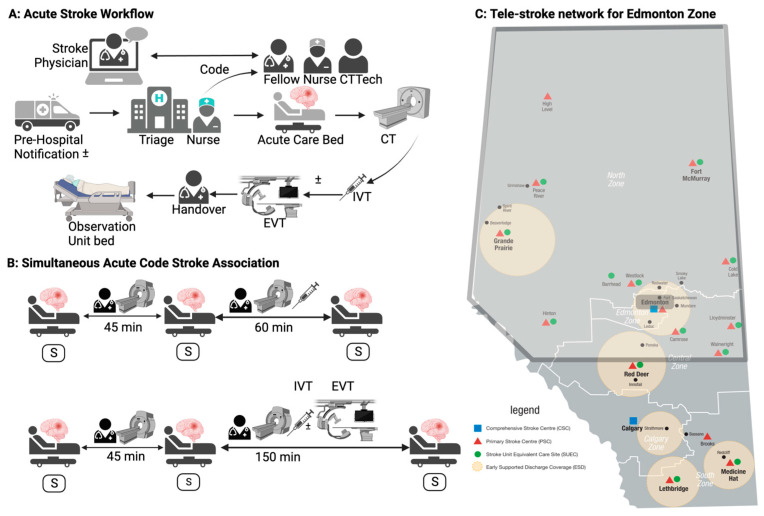
(**A**) Acute stroke workflow: SP, on-call stroke physician; CT tech, computed tomography technician; ED, emergency department; IVT, intravenous thrombolysis; (INR, interventional neuroradiologist/vascular neurosurgeon; INR-N interventional neuroradiology nurse; INR-N Tech, interventional neuroradiology technician; EVT, endovascular thrombectomy. (**B**) Simultaneity in acute code stroke activation (ACSA) definitions; 1: any acute stroke syndrome patient arriving within 60 min of a patient receiving IVT; 2: any acute stroke syndrome patient arriving within 150 min of a patient receiving EVT; 3: any acute stroke syndrome patient arriving within 45 min before the arrival of the patient receiving IVT and/or EVT. (**C**) Tele-stroke network served by the Edmonton Zone.

The acute stroke team at the study hospital consists of an in-house clinical fellow and/or neurology resident and an on-call neurologist. A “code stroke” (i.e. acute stroke activation) is initiated by the triage nurse after the arrival of the patient with acute stroke syndrome. The code stroke message is sent to the stroke nurse, fellow/resident, and imaging technologist. The in-house physician arrives by the bedside within 5 min of the code activation. If a prenotification (<15 min prior to arrival) is available, the in-house physician receives the patient along with the triage nurse. The emergency physician assesses the patient together with the acute stroke team. The on-call neurologist, in addition, is also concurrently on call for 12 tele-stroke sites for patients with acute stroke symptoms (Figure 2).

For each stroke activation, the registry data were used to determine the date of the event, mode of arrival to the hospital, arrival time in triage, and if they had undergone acute reperfusion therapy (IVT and/or EVT) and final diagnosis. In patients receiving either intravenous thrombolysis or endovascular therapy, we extracted the age, sex, National Institute of Health Stroke Scale (NIHSS) on arrival, CT head time, type of reperfusion therapy, time of intravenous thrombolysis, time of groin puncture, and time of reperfusion. The registry data were used to calculate (in minutes) door-to-CT (DTC) time; door-to-needle (DTN) time; door-to-groin puncture (DTP) time; and door-to-reperfusion (DTR) time. The vascular risk factor profile (hypertension, diabetes, dyslipidemia, coronary artery disease, ever a smoker, prior stroke, and functional disability prior to the current stroke) was also extracted from the patients’ charts. 

### 2.3. Study Definitions

For the present study, we defined simultaneity as ACSA 45 min prior to any patient receiving intravenous thrombolysis (IVT) and/or endovascular thrombectomy (EVT); ACSA within 60 min of any patient receiving IVT and ACSA within 150 min of any patient receiving EVT (Figure 2). This is based on the Canadian Triage and Acuity Scale (CTAS) associated workload times, mean local DTN and mean local DTP times. The CTAS is a five-level triage scale implemented since 1999 for categorizing the acuity of a clinical event in a patient presenting to ER [14]. Stroke is classified as level 2, an emergent condition that is a potential threat to life, limb or function and the ideal physician assessment time is <15 min. According to the POWER study, the mean time for the physician to treat patients with CTAS level 2 is 38.9 min (Supplementary Table S1) [15]. Another study focused on the emergency department mean physician treatment time per patient for emergency severity index triage level 1–2 is 76 min and level 3 is 48 min [16]. The mean local DTN is 35 min [17] and the additional workload includes charting and communication with the admitting services and other stakeholders. To further categorize the grading of simultaneity, a system was developed; grade 1 was defined as two patients presenting simultaneously according to the above criteria, grade 2 was defined as three patients presenting simultaneously according to the criteria and grade 3 was defined as > 3 patients presenting simultaneously according to the criteria.

### 2.4. Outcome Measures

Our primary workflow metric-related outcomes measure in patients who received acute reperfusion therapies was DTC (min) and secondary workflow metric-related outcome measures were DTN (min), DTP (min) and DTR (min). Our primary functional outcome measures in patients who received acute reperfusion therapies were the length of hospitalization in an acute care hospital, mortality at 90 days, and home time (HT) at 90 days. Home time has been used as a surrogate marker for functional outcome [18]. The HT was computed based on the number of days the patient spent back at home, 90 days after the stroke onset. 

### 2.5. Sample Size 

In this observational study, we estimated that a 5 min difference in DTC between simultaneous and non-simultaneous would be clinically meaningful, and we expected a standard deviation of 4. Thus, a sample of 198 in each group will achieve a power of 80% and a level of significance of 5%. We aim to assess a 20-month period which will give us an adequate sample size according to our annual IVT and EVT procedures. 

### 2.6. Statistical Analyses 

The patients who received reperfusion therapies were divided into two groups (simultaneous and non-simultaneous) and were further analyzed. Nominal variables (vascular risk factors, sex, IVT, EVT and mortality) were described as proportions in the groups. The skewed continuous variables were described as the median and interquartile range (NIHSS, DTC min, DTN min, DTP min, DTR min, length of hospitalization days and home time days). Age was described as mean and standard deviation. Univariable analysis for nominal variables between simultaneous and non-simultaneous groups was performed using a chi-square test. Univariable analysis between groups for the medians of continuous variables was performed using an independent Student t-test and the Mann–Whitney U test. Age and sex-adjusted stepwise linear regression analysis was performed to assess between-group (non-simultaneous ACSA and simultaneous ACSA) differences. 

A sensitivity analysis was performed to account for variations in workflow metrics in different hospital systems. Setting 1: The higher threshold group (HT) was defined simultaneously as ACSA 60 min prior to any patient receiving intravenous thrombolysis (IVT) and/or endovascular thrombectomy (EVT); ACSA within 75 min of any patient receiving IVT and ACSA within 180 min of any patient receiving EVT. Setting 2: The lower threshold group (LT) was defined simultaneously as ACSA 30 min prior to any patient receiving intravenous thrombolysis (IVT) and/or endovascular thrombectomy (EVT); ACSA within 45 min of any patient receiving IVT and ACSA within 120 min of any patient receiving EVT. Setting 3: The very lower threshold group (VLT) was defined simultaneously as ACSA 30 min prior to any patient receiving intravenous thrombolysis (IVT) and/or endovascular thrombectomy (EVT); ACSA within 30 min of any patient receiving IVT and ACSA within 90 min of any patient receiving EVT. Age and sex-adjusted stepwise linear regression analysis was performed to assess the between-groups differences for each of the 3 settings (non-simultaneous ACSA and simultaneous ACSA). All data were analyzed using STATA 18.0 BE (StataCorp LLC, College Station, TX, USA).

## 3. Results

### 3.1. Baseline Characteristics

A total of 2605 patients were assessed after ACSA during the study period at a mean ± SD of activations of 130.8 ± 17.1/month. A total of 859 (33%) were simultaneous ACSAs, including 67.9% (n = 583) probable stroke patients, 10.7% (n = 92) transient ischemic attack patients and 21.4% (n = 184) with a non-stroke diagnosis. In the non-simultaneous ACSA, 63.6% (n = 1111) were probable stroke patients, 10.5% (n = 183) were patients with transient ischemic attack and 25.9% (n = 452) patients had a non-stroke diagnosis. A total of 545 (20.9%) patients received reperfusion therapy after ACSA and 220 (40.4%) patients were classified as simultaneous ACSA. The baseline characteristics of the simultaneous and non-simultaneous ACSA patients receiving reperfusion therapy were comparable (Table 1). Patients in simultaneous ACSA were more likely to receive endovascular thrombectomy (62.7% versus 53.6%, *p* = 0.03). This relationship was persistent in logistic regression analysis with age, sex and premorbid modified Rankin scale as covariates, patients in the simultaneous group were more likely to undergo EVT (OR 1.5, 95% CI 1.04–2.2). In the simultaneous ACSA patients receiving reperfusion therapy, the severity of simultaneity was grade 1 in 67.3% (n = 148), grade 2 in 26.8% (n = 59) and grade 3 in 5.9% (n = 13).

### 3.2. Workflow Metrics

Median DTC time (min), DTN time (min), DTP time (min) and DTR time (min) were similar in patients with simultaneous ACSA and non-simultaneous ACSA receiving reperfusion therapy. The median DTC time, in minutes, was prolonged in grade 3 simultaneous ACSA (18 (13, 28)) compared to non-simultaneous ACSA (15 (11, 21) β = 0.23, *p* < 0.0001) (Table 2). The DTP time, in minutes, was prolonged in grade 3 simultaneous ACSA (121 (64, 188)) compared to non-simultaneous ACSA (98 (82, 124) β = 0.17, *p* = 0.002). There were no differences in median DTN time (min) and DTR time (min) among different grades. There was no difference in the median home time at 90 days between the simultaneous (58, 0–84.5 days) and non-simultaneous (54, 0–85 days) patients. There was no difference in the hospitalization days and mortality at 90 days between simultaneous and non-simultaneous ACSA (Table 1). 

### 3.3. Threshold Analyses

In the different threshold group comparisons, the proportion of simultaneous ACSA patients with HT was higher and with lower thresholds was lower, as expected. There were no differences in the workflow metrics and home time at 90 days between the non-simultaneous and simultaneous ACSA at different thresholds (Table 3). However, in stepwise age and sex-adjusted linear regression analyses, the DTC time and DTP time were prolonged in grade 3 simultaneous ACSA in the HT, LT and VLT groups (Tables S1–S3).

## 4. Discussion

This study noted simultaneous ACSA in one-third (33%) of all patients assessed by code stroke activation and in 40% of patients receiving acute reperfusion therapies. Grade 3 simultaneous ACSA was associated with prolonged DTC and DTP times. 

Data on simultaneous ACSA are not available. However, we can hypothesize that higher admission volume centers may have increased simultaneity and be subjected to similar workflow metrics effects. An observational study showed that high-IVT-volume centers (7.3%, 200 strokes/year) have lower 7-day stroke-related mortality compared to low-IVT-volume centers (9.5% *p* < 0.0001, <50 strokes/year) [19]. Similar outcomes have also been noted with EVT in stroke patients. In a Dutch study, stroke centers were classified as low volume (<24 IVTs/year), medium volume (25–49 IVTs/year) or high volume (≥50 IVTs/year). The DTN time in the high-volume centers was 8 min faster than in the low-volume centers. Furthermore, patients treated in high-volume centers had a lesser likelihood of symptomatic hemorrhage and mortality [20]. The site case volume affects EVTs and EVT-associated outcomes as well. In an observational study, as the case volume increased for a center, there was a proportionate increase in recanalization and good outcomes and a proportionate decrease in symptomatic intracranial hemorrhage and mortality [21]. A suggested EVT load to improve functional outcomes and reduce mortality was suggested to be more than 23 cases per year [22]. However, a German stroke registry continued to note incremental benefits even at higher volumes of EVTs.

In that study, a total of 5379 patients who underwent EVT were included; centers were classified as high-volume (>180 EVTs/year), medium-volume (135–179 EVTs/year) and low-volume (<135 EVTs/year). High-volume centers had faster DTG, and groin-puncture-to-reperfusion times than the low-volume centers [23]. With 338 IVTs and 312 EVTs over 20 months, our study will be classified as a high-volume center. 

The DTC time was prolonged by a median of 3 min and the DTP time was prolonged by a median of 23 min in grade 3 simultaneous ACSA. This did not affect the outcome measures of mortality and home time. Acute stroke management is a resource-intense endeavor requiring multiple stakeholders from multiple departments (ED, Neurology, Radiology, Nursing). In this study, we were unable to assess the impact of simultaneity on institutional resources, either in terms of human resources or equipment. At the human resource level, simultaneity may act as a stressor, that may lead to decision errors, reduce time spent with patients/family, and reduce emotional self-regulation [24]. Nonetheless, one study showed that although multitasking increases stress experience, it facilitated a professional’s experience of situational awareness [25]. It is worth noting that one-fifth of the ACSA in the current study did not have a stroke diagnosis; however, these patients were still assessed by the stroke team. A coordinated care pathway with an appropriate human resources structure for the acute stroke ED team members may be helpful, particularly the involvement of a stroke nurse practitioner in addition to a stroke physician/fellow/resident, ED physician, stroke nurse, and endovascular therapy nurse. Simultaneous availability of more than one EVT suite in a high-volume center may be challenging. Similarly, access to a CT scanner for simultaneous patients may either require waiting or a transfer of patients to another CT scanner at a different site. One study by Dalsania et al. demonstrated that concurrent demand for EVT is likely to occur in institutions with higher annual patient volumes [10]. A recent Get with the guidelines-Stroke registry observed higher volume EVT sites were associated with a greater likelihood of being discharged home, better functional outcomes, and decreased mortality. However, these beneficial outcomes have a ceiling effect at 110 EVT procedures per year [26].

### Limitations

In this retrospective analysis of a prospective registry at a single high-volume center, all predictors may not have been captured in the database. Particularly the impact of simultaneity on institutional (imaging resources) and human resources (w.r.t. physical and mental stress). The study definitions are institution specific as the DTN and DTG times may vary according to the center. Thus, it would be appropriate to develop the local definition of simultaneity at each site. We used home time at 90 days as our outcome measure as mRS data was not captured in the registry. This is a single-center experience and may not reflect the effects of simultaneity at other busy centers.

## 5. Conclusions

Our study demonstrated that simultaneity in ACSA is frequent in patients receiving acute reperfusion therapies. Although simultaneity was frequent, it did not result in any detrimental effects in patients treated with IVT or EVT. It is important that busy centers evaluate the effects of simultaneity on outcomes locally. Strategies such as improving patient flow, involving nurse practitioners in ED, implementing triage protocols, and optimizing resource allocation may be helpful if simultaneity leads to delays in treatment but this requires further studies. An optimal workflow may help mitigate the clinical and system burden associated with simultaneity. 

## Figures and Tables

**Table 1:** Baseline and outcome characteristics of patients receiving acute reperfusion therapies in the acute code stroke activation cohort.

	Non-Simultaneous ACSA (n = 325)	Simultaneous ACSA (n = 220)	*p* Value
Age, years mean ± SD	70.9 ± 13.7	70.5 ± 14.6	0.4
Female Sex, n (%)	143 (44%)	107 (48.6%)	0.2
Prior disability, n (%)	47 (14.5%)	37 (16.8%)	0.4
Median (IQR) NIHSS	13 (8–18)	13 (8–18)	0.5
**Risk Factors**			
Hypertension, n (%)	209 (64.3%)	141 (64.1%)	0.9
Diabetes Mellitus, n (%)	72 (22.1%)	53 (24.1%)	0.6
Dyslipidemia, n (%)	125 (38.5%)	81 (36.8%)	0.6
Atrial Fibrillation, n (%)	53 (16.3%)	44 (20%)	0.3
Coronary artery Disease, n (%)	52 (16%)	32 (14.5%)	0.6
Smoker, n (%)	64 (19.7%)	41 (18.6%)	0.4
Prior Stroke, n (%)	65 (20%)	35 (15.9%)	0.2
**Acute Reperfusion Therapies**			
Intravenous Thrombolysis, n (%)	210 (64.6%)	128 (58.2%)	0.1
Endovascular Thrombectomy, n (%)	174 (53.6%)	138 (62.7%)	0.03
**Short-Term Outcome**			
Median (IQR) length of Hospital Stay, Days	13 (4, 45)	17 (4, 49)	0.6
Median Home Time, Days	54 (0, 85)	58 (0, 84.5)	0.7
Mortality at 90 days, n (%)	63 (19.4%)	38 (17.2%)	0.5

ACSA, acute code stroke activation; NIHSS, National Institute of Health Stroke Scale.

**Table 2:** Median workflow times and home time in patients with simultaneous and non-simultaneous acute code stroke activation receiving reperfusion therapy.

Median (IQR) Workflow Times	Non-Simultaneous ACSA	Simultaneous ACSA		
		Grade 1	Grade 2	Grade 3
DTC, min	15 (11, 21) n = 325	16 (11, 21) n = 148	15 (10.5, 22.5) n = 59	18 (13–28) * n = 13
DTN, min	39 (30, 55) n = 210	39 (30, 50.5) n = 92	39 (30, 57) n = 31	53 (30–56) n = 5
DTP, min	98.5 (82, 124) n = 174	101 (83, 127) n = 89	89.5 (69, 114) n = 38	121 (64, 188) * n = 11
DTR, min	149 (119, 177) n = 174	141 (114, 184) n = 89	119.5 (103, 156) n = 38	155 (108.5, 224.5) n = 11
Home Time, days	54 (0, 85) n = 325	54.5 (0, 84) n = 148	66 (2, 85.5) n = 59	61 (29, 84) n = 13

ACSA, acute code stroke activation; DTC, door-to-CT time, min; DTN, door-to-needle time, min; DTP, door-to-groin puncture time, min; DTR, door-to-reperfusion time, min; *, *p* < 0.05.

**Table 3:** Workflow and outcome metrics in different threshold groups.

Median (IQR)	Study Definition		Higher Threshold		Low Threshold		Very Low Threshold	
	NS-ACSA (n = 325)	S-ACSA (n = 220)	NS-ACSA (n = 291)	S-ACSA (n = 254)	NS-ACSA (n = 364)	S-ACSA (n = 181)	NS-ACSA (n = 405)	S-ACSA (n = 140)
DTC (min)	15 (11, 21)	16 (11, 22.5)	15 (11, 21)	16 (11, 23)	15 (11, 21)	16 (11, 23)	15 (11, 21)	17 (12, 25)
DTN (min)	39 (30, 55)	39 (30.5, 53)	39 (29, 55)	39 (30.5, 52)	38 (30, 55)	40 (31, 53)	38 (30, 52)	40 (31, 53)
DTP (min)	98.5 (82, 124)	98.5 (79, 125)	97 (811, 123)	101 (80, 126)	98.5 (81, 124.5)	98.5 (79.5, 125)	98.5 (81, 122.5)	98.5 (79.5, 130)
DTR (min)	149 (119, 177)	140.5 (110, 167.5)	147 (121, 173)	141 (112, 168)	147 (120, 173.5)	140 (111, 161)	147 (116, 172)	141.5 (113, 173)
Home Time (days)	54 (0, 85)	58 (0, 84.5)	55 (0, 85)	56 (0, 84)	55 (0, 85)	57 (0, 84)	55 (0, 85)	61 (3.5, 84.5)

NS-ACSA, Non-simultaneous acute code stroke activation; S-ACSA, simultaneous acute code stroke activation; DTC, door-to-CT time, min; DTN, door-to-needle time, min; DTP, door-to-groin puncture time, min; DTR, door-to-reperfusion time, min.

## Data Availability

Data will be available to interested researchers, please contact the corresponding author.

## Supplementary Materials

The following supporting information can be downloaded at: https://www.mdpi.com/article/10.3390/neurosci5030023/s1, Table S1: Distribution of workflow metrics and 90-day home time in the higher threshold category. Table S2: Distribution of workflow metrics and 90-day home time in the lower threshold category. Table S3: Distribution of workflow metrics and 90-day home time in the Very lower threshold category.

## References

[1] Saver J.L., Goyal M., van der Lugt A., Menon B.K., Majoie C.B.L.M., Dippel D.W., Campbell B.C., Nogueira R.G., Demchuk A., Tomasello A. (2016). Time to Treatment With Endovascular Thrombectomy and Outcomes From Ischemic Stroke: A Meta-analysis. JAMA.

[2] Jovin T.G., Nogueira R.G., Lansberg M.G., Demchuk A.M., Martins S.O., Mocco J., Ribo M., Jadhav A.P., Ortega-Gutierrez S., Hill M.D. (2022). Thrombectomy for anterior circulation stroke beyond 6 h from time last known well (AURORA): A systematic review and individual patient data meta-analysis. Lancet.

[3] Alemseged F., Nguyen T.N., Alverne F.M., Liu X., Schonewille W.J., Nogueira R.G. (2023). Endovascular Therapy for Basilar Artery Occlusion. Stroke.

[4] Janssen P.M., Venema E., Dippel D.W.J. (2019). Effect of Workflow Improvements in Endovascular Stroke Treatment. Stroke.

[5] Ding Y., Gao F., Ji Y., Zhai T., Tong X., Jia B., Wu J., Wu J., Zhang Y., Wei C. (2021). Workflow Intervals and Outcomes of Endovascular Treatment for Acute Large-Vessel Occlusion During On-Vs. Off-hours in China: The ANGEL-ACT Registry. Front. Neurol..

[6] Heran M., Lindsay P., Gubitz G., Yu A., Ganesh A., Lund R., Arsenault S., Bickford D., Derbyshire D., Doucette S. (2022). Canadian Stroke Best Practice Recommendations: Acute Stroke Management, 7th Edition Practice Guidelines Update, 2022. Can. J. Neurol. Sci./J. Can. Sci. Neurol..

[7] Chen C.-H., Tang S.-C., Tsai L.-K., Hsieh M.-J., Yeh S.-J., Huang K.-Y., Jeng K.-Y. (2014). Stroke Code Improves Intravenous Thrombolysis Administration in Acute Ischemic Stroke. PLoS ONE..

[8] Reznek M.A., Murray E., Youngren M.N., Durham N.T., Michael S.S. (2017). Door-to-Imaging Time for Acute Stroke Patients Is Adversely Affected by Emergency Department Crowding. Stroke.

[9] Kelen G.D., Wolfe R., D’Onofrio G., Mills A.M., Diercks D., Stern S.A., Wadman M.C., Sokolove P.E. (2021). Emergency Department Crowding: The Canary in the Health Care System. NEJM Catal..

[10] Dalsania A.K., Kansagra A.P. (2019). Simultaneous patient presentation for endovascular thrombectomy in acute ischemic stroke. J. Neurointerv. Surg..

[11] Buck B.H., Akhtar N., Alrohimi A., Khan K., Shuaib A. (2021). Stroke mimics: Incidence, aetiology, clinical features and treatment. Ann. Med..

[12] Moskop J.C., Sklar D.P., Geiderman J.M., Schears R.M., Bookman K.J. (2009). Emergency Department Crowding, Part 1—Concept, Causes, and Moral Consequences. Ann. Emerg. Med..

[13] Ding R., McCarthy M.L., Desmond J.S., Lee J.S., Aronsky D., Zeger S.L. (2010). Characterizing Waiting Room Time, Treatment Time, and Boarding Time in the Emergency Department Using Quantile Regression. Acad. Emerg. Med..

[14] Bullard M.J., Musgrave E., Warren D., Unger B., Skeldon T., Grierson R., van der Linde E., Swain J. (2017). Revisions to the Canadian Emergency Department Triage and Acuity Scale (CTAS) Guidelines 2016. Can. J. Emerg. Med..

[15] Dreyer J.F., McLeod S.L., Anderson C.K., Carter M.W., Zaric G.S. (2009). Physician workload and the Canadian Emergency Department Triage and Acuity Scale: The Predictors of Workload in the Emergency Room (POWER) Study. CJEM.

[16] Wrede J., Wrede H., Behringer W. (2020). Emergency Department Mean Physician Time per Patient and Workload Predictors ED-MPTPP. J. Clin. Med..

[17] Kamal N., Jeerakathil T., Stang J., Liu M., Rogers E., Smith E.E., Demchuk A., Siddiqui M., Mann B., Bestard J. (2020). Provincial Door-to-Needle Improvement Initiative Results in Improved Patient Outcomes Across an Entire Population. Stroke.

[18] Quinn T.J., Dawson J., Lees J.S., Chang T.-P., Walters M.R., Lees K.R. (2008). Time Spent at Home Poststroke. Stroke.

[19] Saposnik G., Baibergenova M.A., O’Donnell M., Hill M.D., Kapral M.K., Hachinski V. (2007). Hospital volume and stroke outcome Does it matter?. Neurology.

[20] Groot A.E., van Schaik I.N., Visser M.C., Nederkoorn P.J., Limburg M., Aramideh M., de Beer F., Zwetsloot C.P., Halkes P., de Kruijk J. (2016). Association between i.v. thrombolysis volume and door-to-needle times in acute ischemic stroke. J. Neurol..

[21] Kim B.M., Baek J.H., Heo J.H., Kim D.J., Nam H.S., Kim Y.D. (2019). Effect of Cumulative Case Volume on Procedural and Clinical Outcomes in Endovascular Thrombectomy. Stroke.

[22] Shim D.H., Kim Y., Roh J., Kang J., Park K.P., Cha J.K., Baik S.K., Kim Y. (2020). Hospital volume threshold associated with higher survival after endovascular recanalization therapy for acute ischemic stroke. J. Stroke.

[23] Hahn M., Gröschel S., Tanyildizi Y., Brockmann M.A., Gröschel K., Uphaus T. (2022). The Bigger the Better? Center Volume Dependent Effects on Procedural and Functional Outcome in Established Endovascular Stroke Centers. Front. Neurol..

[24] West C.P., Dyrbye L.N., Shanafelt T.D. (2018). Physician burnout: Contributors, consequences and solutions. J. Intern. Med..

[25] Augenstein T., Schneider A., Wehler M., Weigl M. (2021). Multitasking behaviors and provider outcomes in emergency department physicians: Two consecutive, observational and multi-source studies. Scand. J. Trauma Resusc. Emerg. Med..

[26] Nogueira R.G., Haussen D.C., Smith E.E., Sun J.L., Xian Y., Alhanti B., Blanco R., Mac Grory B., Doheim M.F., Bhatt D.L. (2023). Higher Procedural Volumes Are Associated with Faster Treatment Times, Better Functional Outcomes, and Lower Mortality in Patients Undergoing Endovascular Treatment for Acute Ischemic Stroke. Ann. Neurol..

