# Supplementary material for: Workflow and Short-Term Functional Outcomes in Simultaneous Acute Code Stroke Activation and Stroke Reperfusion Therapy
Source: NeuroSci. 2024 Aug 22;5(3):291–300. doi: 10.3390/neurosci5030023 (PMC11469737; doi:10.3390/neurosci5030023)
Supplement: Supplementary file 1 [file neurosci-05-00023-s001.zip › neurosci-3140866-supplementary.pdf]

Supplementary Tables

Supplementary Table S1: Distribution of workflow metrics and 90-day home time in the higher threshold category

Median Workflow Times	Non-Simultaneous ACSA (n=291, 53.4%)	Simultaneous ACSA (n=254, 46.6%)		
		Grade 1 (n=166, 30.4%)	Grade 2 (n=67, 12.3%)	Grade 3 (n=21, 3.9%)
DTC, min	15 (11,21)	16(11,22)	16(11,23)	18 (13,28) *
DTN, min	39 (29,55)	38.5(30,48)	39(32,57)	45(30,56)
DTP, min	97 (811,123)	104.5 (84,128)	86(67.5, 109.5)	121 (66, 185) **
DTR, min	147 (121,173)	147 (118.5,173)	120 (103,149)	147 (103,192)
Home Time, days	55 (0,85)	48.5(0,84)	64(0,85)	68(29,86)

ACSA, Acute code stroke activation; DTC, Door-to-CT time, min; DTN, Door-to-needle time, min; DTP, Door-to-groin puncture time, min; DTR, Door-to-reperfusion time, min; *, p<0.0001; ** p=0.007

Supplementary Table S2: Distribution of workflow metrics and 90-day home time in the lower threshold category

Median Workflow Times	Non-Simultaneous ACSA (n=364, 66.8%)	Simultaneous ACSA (n=181, 33.2%)		
		Grade 1 (n=125, 22.9%)	Grade 2 (n=47, 8.6%)	Grade 3 (n=9, 1.6%)
DTC, min	15 (11,21)	16 (11,21)	17(11,28)	18(13,30) *
DTN, min	38 (30,55)	39.5 (30.5,48)	44.5 (38,57)	43(28.5,58.5)
DTP, min	98.5 (81, 124.5)	99.5 (82, 126)	97 (77, 117)	153 (87, 192.5) **
DTR, min	147 (120,173.5)	141(113, 162)	123 (106, 152)	144.5 (100, 224.5)
Home Time, days	55 (0,85)	52 (0,83)	66(0,86)	61(29,84)

ACSA, Acute code stroke activation; DTC, Door-to-CT time, min; DTN, Door-to-needle time, min; DTP, Door-to-groin puncture time, min; DTR, Door-to-reperfusion time, min
*, $p<0.0001$; ** $p<0.0001$

Supplementary Table S3: Distribution of workflow metrics and 90-day home time in the Very lower threshold category

Median Workflow Times	Non-Simultaneous ACSA (n=405, 74.3%)	Simultaneous ACSA (n=140, 25.7%)		
		Grade 1 (n=106, 19.5%)	Grade 2 (n=27, 4.9%)	Grade 3 (n=7, 1.3%)
DTC, min	15 (11, 21)	16 (11, 22)	17 (13, 28)	25 (14, 35) *
DTN, min	38 (30, 52)	40 (31, 53)	46 (38, 61)	43(28.5, 58.5)
DTP, min	98.5 (81, 122.5)	95.5 (80, 130)	98 (78, 118)	156 (112, 194) **
DTR, min	147 (116,172)	141 (111,173)	139 (122, 160)	144.5 (120, 220)

Home Time, days	55 (0, 85)	55 (0,85)	68 (13, 84)	61 (29, 86)

ACSA, Acute code stroke activation; DTC, Door-to-CT time, min; DTN, Door-to-needle time, min; DTP, Door-to-groin puncture time, min; DTR, Door-to-reperfusion time, min
*, p<0.0001; ** p<0.0001
